# TM4SF1 mediates YAP/TAZ regulation of coronary artery formation

**DOI:** 10.1038/s41598-026-57461-x

**Published:** 2026-06-11

**Authors:** Yuan Fang, Jinling Dong, Hongyu Su, Weihao Xue, Jing Chen, Bin Zhou, Hao Hu, Yidong Wang

**Affiliations:** 1 The Institute of Cardiovascular Sciences, School of Basic Medical Sciences, Key Laboratory of Environment and Genes Related to Diseases, Ministry of Education, Xi’an Jiaotong University, Xi’an, Shaanxi China; 2 Department of Cardiology, The Second Affiliated Hospital of Wenzhou Medical University, Wenzhou, Zhejiang China; 3 Shaanxi Stem Cell Application Engineering Research Center, Shaanxi Jiuzhou Biomedical Science and Technology Group, Xi’an, Shaanxi China; 4 Department of Pediatrics, The University of Chicago, Chicago, IL USA; 5 Department of Pharmacology, School of Basic Medical Sciences, Xi’an Jiaotong University, Xi’an, Shaanxi China; 6 Department of Cardiology, Wenling First People’s Hospital, The Affiliated Hospital of Wenzhou Medical University, Wenling, Zhejiang China

**Keywords:** Coronary artery development, Angiogenesis, Endocardium, YAP/TAZ, TM4SF1, Cardiology, Cell biology, Developmental biology

## Abstract

Although the role of YAP/TAZ in mediating VEGF-driven angiogenesis is well established, their downstream effectors in coronary artery development remain elusive. In this study, we identify TM4SF1 as a novel downstream target of YAP/TAZ in coronary endothelial cells. Conditional deletion of YAP/TAZ in the endocardial lineage resulted in embryonic lethality due to congenital heart defects, including ventricular noncompaction. Mutant embryos exhibited severe coronary vessel defects, including the absence of major arteries. Loss of YAP/TAZ significantly inhibited endothelial cell proliferation and coronary angiogenesis. Transcriptomic profiling revealed that YAP/TAZ deficiency downregulated the expression of TM4SF1, a known regulator of angiogenesis, in the endocardium and coronary endothelium. Consistently, YAP/TAZ were required for VEGF-induced upregulation of TM4SF1 in endothelial cells. Moreover, YAP/TAZ-mediated upregulation of TM4SF1 was essential for VEGF-driven angiogenesis in HUVECs. Together, these findings indicate that TM4SF1 functions downstream of YAP/TAZ to regulate endothelial angiogenic processes, suggesting a potential role for TM4SF1 in coronary artery development.

**Supplementary Information:**

The online version contains supplementary material available at 10.1038/s41598-026-57461-x.

## Introduction

The coronary artery is responsible for delivering oxygen and nutrients to cardiomyocytes in both fetal and postnatal life^1,2^. Coronary arterial endothelial cells develop through a multistep program that originates from progenitor cells arising from the sinus venosus endothelium^3–5^ and ventricular endocardium^4,6,7^ with minor contributions coming from the epicardium^8^. These precursors invade the myocardial wall, coalesce into a primitive endothelial plexus via vasculogenesis, and subsequently undergo arterial specification and maturation by recruiting smooth muscle cells and pericytes^9–11^. Recent studies have demonstrated that the arterial identity of nascent coronary vessels is reinforced by NOTCH^12,13^, VEGF^5,14^, Ino80^15^, and CXCL12/CXCR4 signaling^16,17^, while mechanical forces and hemodynamic flow further sculpt the final coronary tree^18^. Developmental abnormalities in the coronary artery often lead to congenital heart defects, including ventricular noncompaction during development and increase the risk of ischemic heart diseases later in life^19^.

The Hippo pathway is a master regulator of organ size across species^20,21^. YAP (Yes-associated protein) and its paralogue TAZ are the core transcriptional co-activators downstream of the Hippo pathway. When Hippo signaling is inactive, de-phosphorylated YAP/TAZ translocate to the nucleus, bind TEAD transcription factors, and drive gene programs that control proliferation, migration, differentiation, and survival^22–24^. Mouse genetic studies have established the critical role of YAP in heart development^24^. *Heallen et al.* have shown that Hippo-deficient mouse embryos exhibit cardiac overgrown with increased cardiomyocyte proliferation^25^. Mechanistically, attenuated Hippo signaling promotes nuclear accumulation of YAP, where it enhances Wnt signaling through direct interaction with β-catenin^25^. Consistently, *Xin et al.* reported that cardiac-specific knockout of *Yap* using Nkx2.5-Cre disrupts myocardial development due to reduced cardiomyocyte proliferation, resulting in embryonic lethality by embryonic day 10.5 (E10.5)^26^. YAP1 is essential for early heart valve development by promoting endothelial to mesenchymal transformation^27^. *Singh et al.* demonstrated that selective deletion of YAP/TAZ in the epicardium inhibits epicardial cell proliferation and differentiation, leading to defective coronary artery development^28^. In addition, YAP/TAZ in the endocardium critically regulates myocardial growth via Nrg1-mediated paracrine signaling during early heart development^29^. However, the cell-autonomous role of endothelial YAP/TAZ in coronary artery development remains undefined.

YAP/TAZ are well-established regulators of developmental and pathological angiogenesis^30^. *Wang et al.* demonstrated that nuclear translocation of YAP/TAZ transduces VEGF signaling into a specific transcriptional program required for a full angiogenic response^31^. Nevertheless, the downstream targets of YAP/TAZ in mediating VEGF-driven coronary angiogenesis remain to be elucidated. In this study, by combining endocardial-specific YAP/TAZ knockout mice, transcriptomic profiling, and in vitro angiogenesis assays, we identify TM4SF1 as a novel downstream mediator of YAP/TAZ in regulation of coronary artery development.

## Results

### Deletion of *Yap/Taz* in endocardial lineage impedes heart development and causes embryonic lethality

**Fig. 1:**
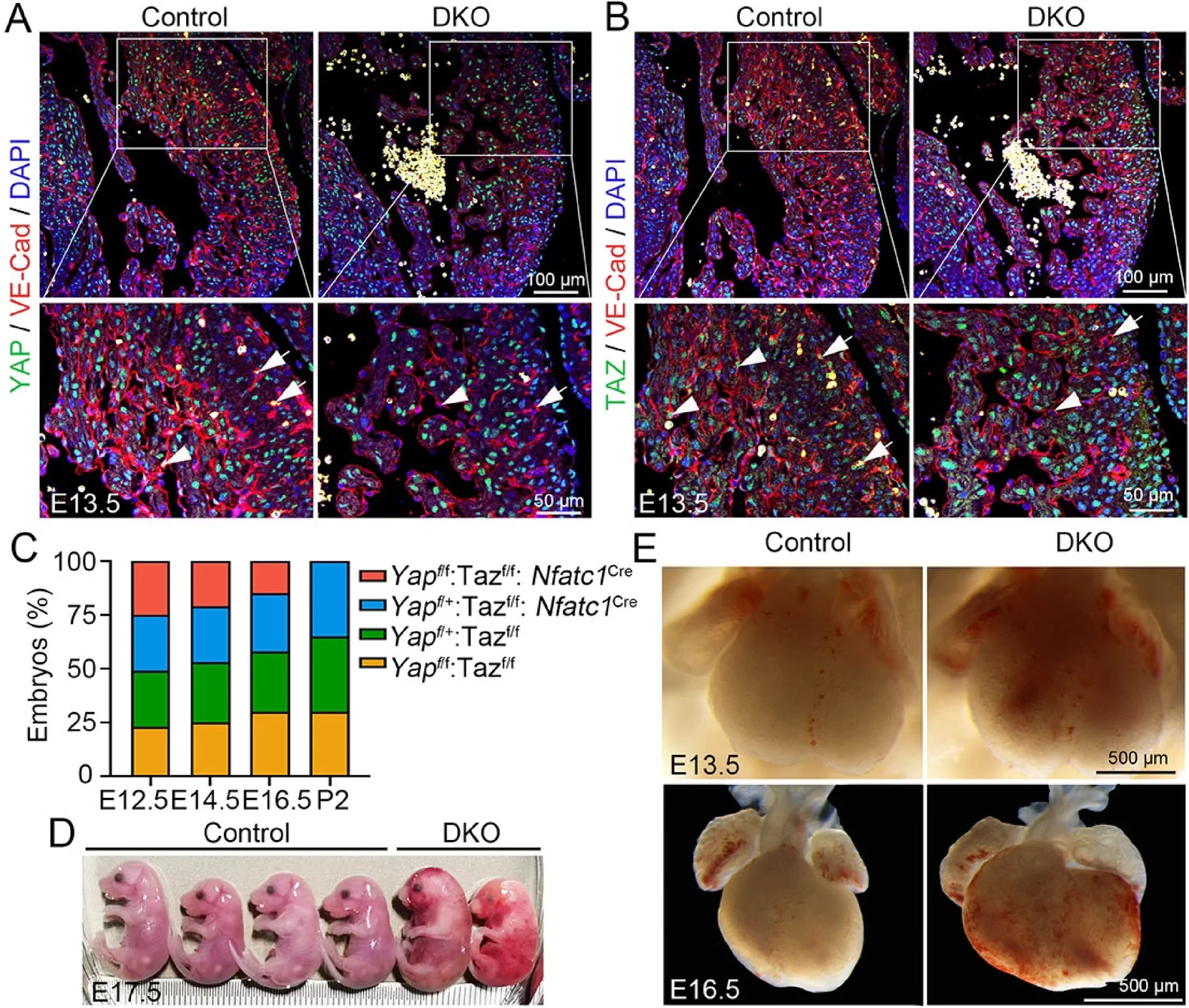
Loss of YAP/TAZ in endocardial lineage impedes heart development and causes embryonic lethality. (**A**, **B**) Immunofluorescence staining confirms selective loss of YAP and TAZ expression in endocardial cells (arrowheads) and coronary arterial endothelial cells (arrows) of DKO embryos (*Yap*^f/f^:*Taz*^f/f^: *Nfatc1*^Cre^) compared to littermate controls (*Yap*^f/f^:*Taz*^f/f^ or *Yap*^f/+^:*Taz*^f/f^) at embryonic day 13.5 (E13.5). Endocardial and endothelial cells are labeled by immunostaining of VE-Cadherin (VE-Cad). (**C**) Genotype distribution of embryos from crosses between *Yap*^f/+^:*Taz*^f/f^: *Nfatc1*^Cre^ and *Yap*^f/f^:*Taz*^f/f^ mice at indicated stages. (**D**, **E**) Gross morphology of whole embryos (**D**) and hearts (**E**) at indicated stages.

By crossing *Yap*^f/f^*/Taz*^f/f^ mice with *Nfatc1*^cre^ mice^6^, we generated a conditional knockout mouse line (*Yap*^f/f^*/Taz*^f/f^: *Nfatc1*^cre^, hereafter referred to as DKO) in which *Yap/Taz* were selectively deleted in endocardial lineage since embryonic day 8.0 (E8.0), while their littermates (*Yap*^f/f^*/Taz*^f/f^ or *Yap*^f/+^:*Taz*^f/f^) were used as controls. Immunofluorescence staining revealed that the expression YAP/TAZ in E13.5 DKO embryos was selectively ablated in endocardial and coronary endothelial cells but not in cardiomyocytes compared to controls (Fig. 1A,B and Suppl. Fig. 1). The DKO embryos exhibited perinatal lethality (Fig. 1C) and edema at E17.5 (Fig. 1D). The hearts of DKO embryos were similar as those of controls at E13.5 but progressed to dilated, hemorrhagic phenotypes by E16.5, indicating heart failure (Fig. 1E). These results indicate that *Yap/Taz* in endocardial lineage is essential for heart development and embryo survival.

### *Yap/Taz* loss leads to ventricular noncompaction

**Fig. 2:**
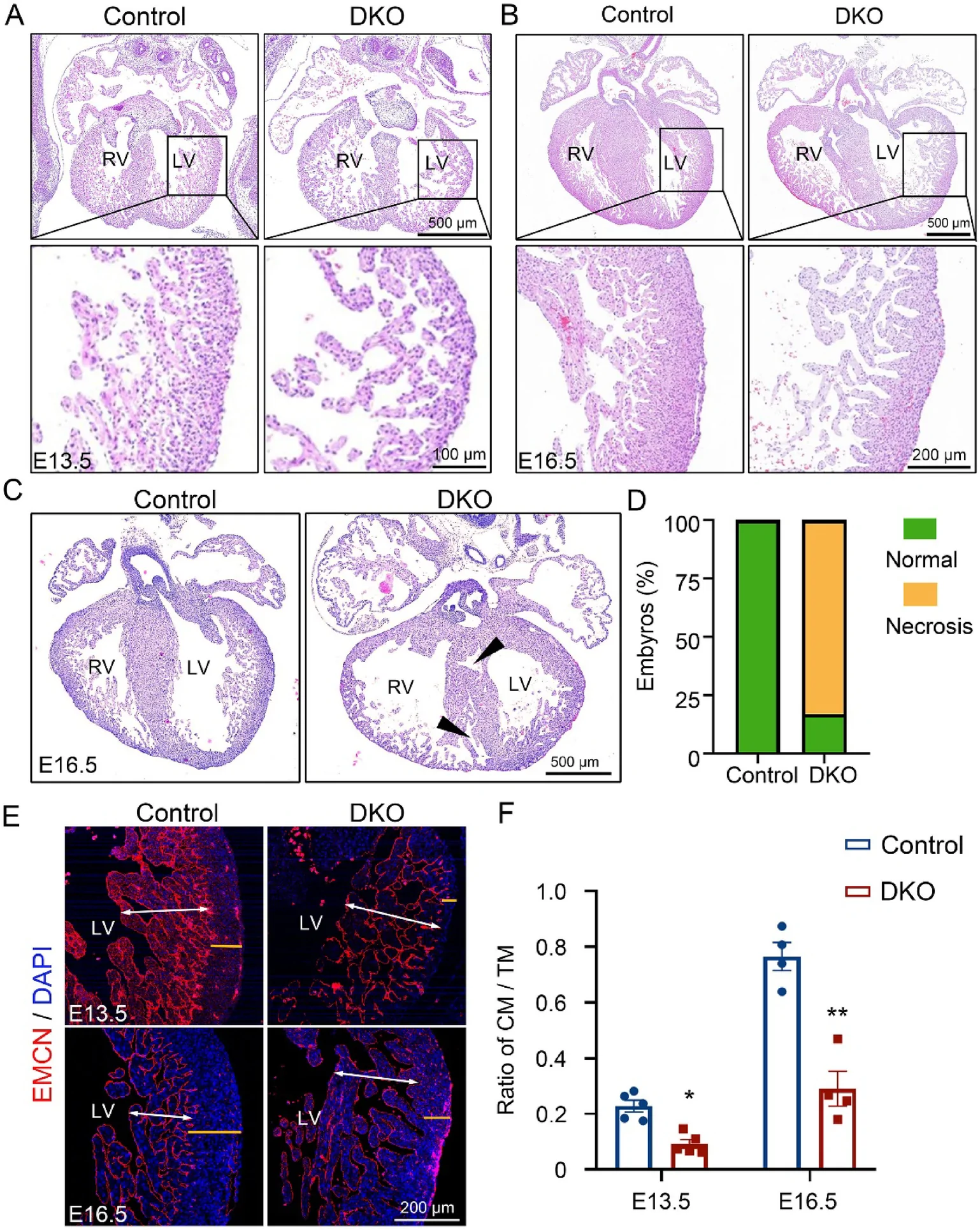
YAP/TAZ deficiency causes ventricular noncompaction. (**A**–**D**) Representative hematoxylin and eosin (H&E) staining of heart sections from control and DKO embryos at indicated stages (**A**–**C**). Arrowheads indicate necrotic lesions within the ventricular septum (VS) of DKO embryos (**C**). The incidence of this phenotype at E16.5 is shown (**D**). (**E**, **F**) Immunostaining for EMCN marking the trabecular myocardium (TM) and compact myocardium (CM) in control and DKO embryos at indicated stages. The widths of the TM (indicated by white double‑headed arrows) and the CM (indicated by yellow lines) were quantified and are presented as the CM/TM ratio (**F**). LV, left ventricle; RV, right ventricle. *n* = 4–6/group. *p < 0.05, **p < 0.01 by unpaired Student’s *t*-test.

To precisely characterize the structural alterations, we subjected cardiac specimens from control and DKO embryos at different developmental stages to comprehensive histological evaluation via hematoxylin and eosin (H&E) staining. Compared with controls, the DKO embryos had much thinner compaction myocardium in both left and right ventricle chambers at E13.5 and E16.5 (Fig. 2A,B). This finding is consistent with the hypoplastic ventricular wall phenotype previously reported in *Yap*/*Taz* knockout embryos generated using the same Cre line as the present study^29^. Notably, membranous ventricular septal defects were observed in 50% (3/6) of DKO embryos at E13.5 (Fig. 2A and Suppl. Fig. 2), and necrotic cavities within the interventricular septum were present in 83% (5/6) at E16.5 (Fig. 2C,D). In addition, immunofluorescence staining of EMCN was performed to label the endocardium. Compared with controls, DKO embryos exhibited a significantly thickened trabecular layer and a correspondingly thinned compact myocardium in both chambers, as reflected by a reduced compact‑to‑trabecular myocardial thickness ratio (Fig. 2E,F and Suppl. Fig. 3). These findings indicate that deletion of *Yap/Taz* in endocardial lineage results in ventricular noncompaction phenotype, indicative of hypoxic–ischemic compromise.

### *Yap/Taz* deficiency disrupts coronary artery formation

**Fig. 3:**
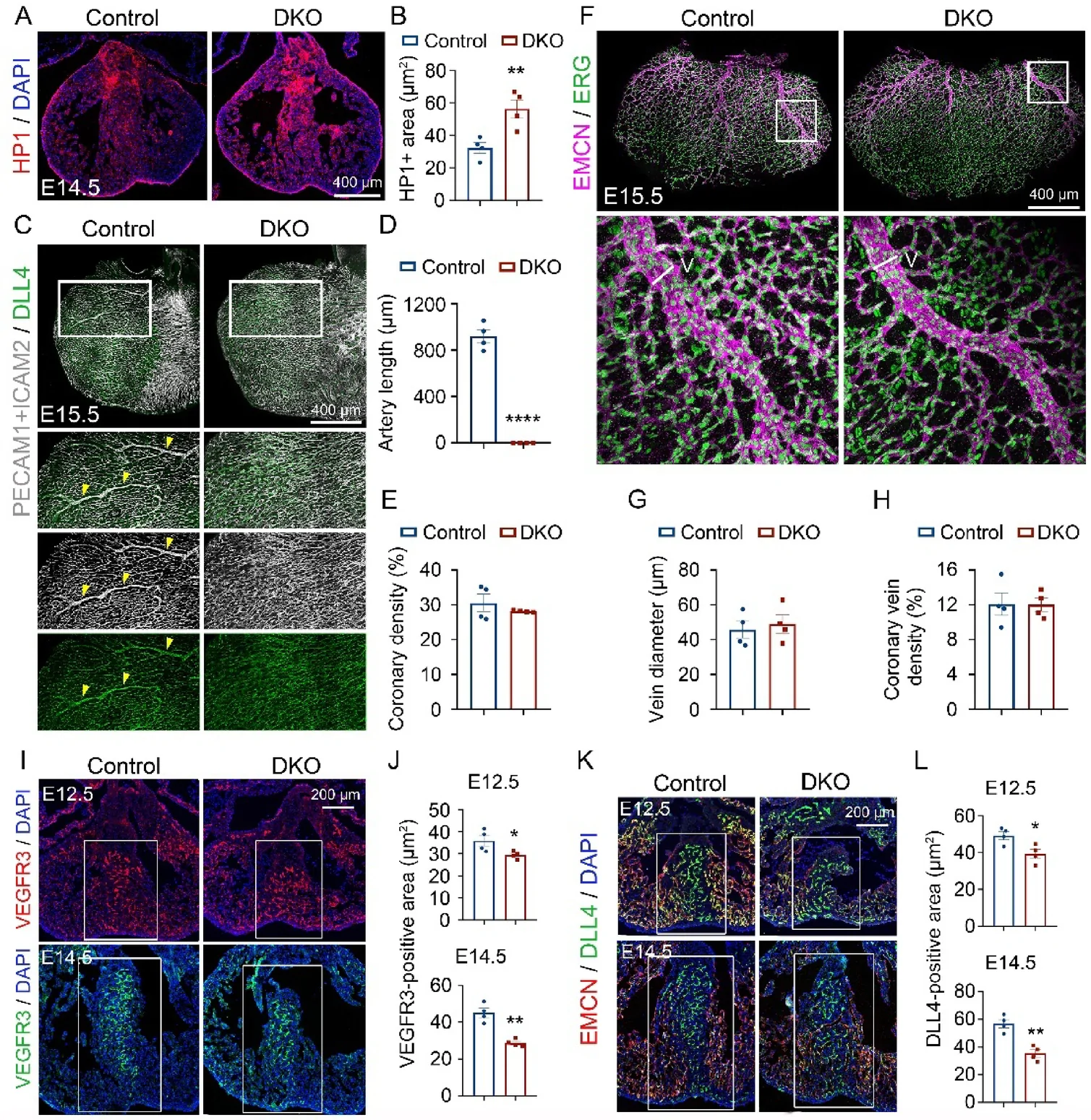
Loss of YAP/TAZ disrupts coronary artery formation. (**A**, **B**) Hypoxia probe (HP1) staining on heart sections from control and DKO embryos. Quantification of hypoxic area is shown (**B**). *n* = 4/group. (**C**–**H**) Whole-mount staining of E15.5 hearts with antibodies against DLL4 (arterial endothelial marker), PECAM1 and ICAM2 (pan-endothelial markers), EMCN (venous endothelial marker) and ERG (endothelial nuclei marker). The lower panels showing the high magnifications of the boxed regions. The yellow arrowheads indicate coronary arteries. Bar charts showing quantifications for artery length (**D**), coronary density (**E**), vein diameter (**G**) and coronary vein density (**H**). *n* = 4/group. (**I**, **J**) Representative images and corresponding quantifications for VEGFR3 (tip cell marker) immunostaining on heart sections at E12.5 and E14.5. *n* = 4/group. (**K**, **L**) Co-immunostaining of DLL4 (tip cell marker) and EMCN on heart sections at indicated stages. Bar charts showing quantifications for Dll4-postive area (**L**). *n* = 4/group. *p < 0.05, **p < 0.01, ****p < 0.0001 by unpaired Student’s *t*-test.

Subsequently, we evaluated coronary circulation in E14.5 control and DKO embryos by using hypoxia probe 1 (HP1). In line with the ventricular noncompaction phenotype, DKO hearts exhibited severe hypoxia (Fig. 3A,B), indicating coronary artery defects. Indeed, whole mount staining of hearts from E15.5 embryos with PECAM1 plus ICAM2 (marked all vessels) and DLL4 (marked coronary artery) antibodies revealed that the DKO embryos had severe coronary artery defects, evidenced by absence of major coronary arteries (Fig. 3C–E and Suppl. Fig. 4). In contrast, the coronary veins labeled by EMCN were comparable between control and DKO embryos (Fig. 3F–H and Suppl. Fig. 4). Consistent with the observations made by whole mount staining, the DKO embryos had significantly less active angiogenic endothelial cells marked by either DLL4 or VEGFR3 compared to controls (Fig. 3I–L). Collectively, these results demonstrate that *Yap/Taz* in endocardial lineage is essential for coronary artery formation by controlling proper angiogenesis for arteriogenesis.

### *Yap/Taz* deficiency inhibits endothelial cell proliferation

**Fig. 4:**
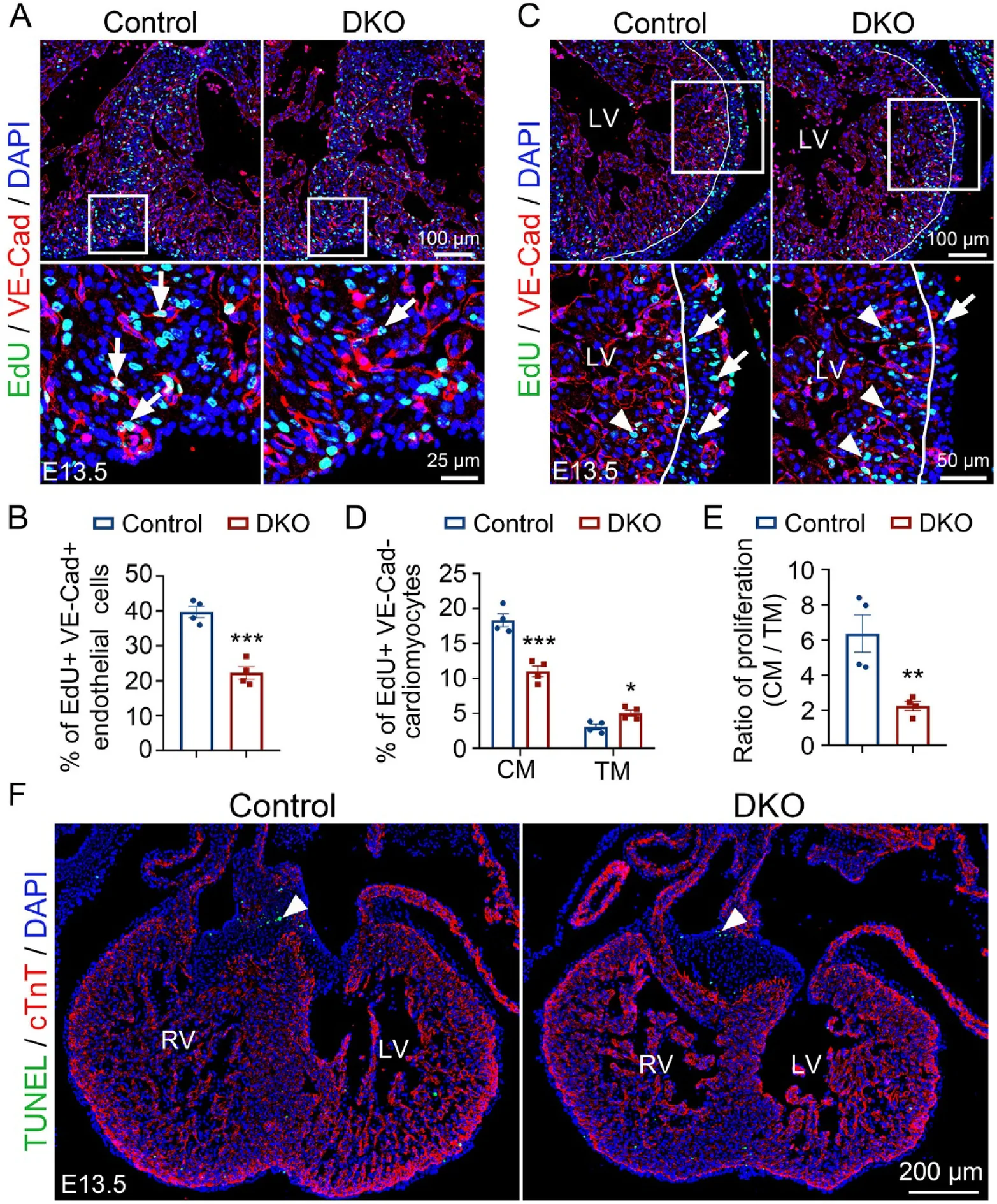
Loss of YAP/TAZ impairs cell proliferation. (**A**–**E**) Representative immunofluorescence images (**A**, **C**) and quantitative analysis of proliferating endothelial cells marked by VE‑cadherin (VE-Cad) (**B**, arrows) and cardiomyocytes (**D**) on heart sections from E13.5 control and DKO embryos. Cardiomyocyte proliferation was quantified separately in the compact myocardium (CM) and trabecular myocardium (TM) (**D**). The ratio of cell proliferation in CM to that in TM is shown in (**E**). *n* = 4/group. (**F**) Representative images showing TUNEL labeling of apoptotic cells (arrowheads) in atrioventricular canal cushions from E13.5 control and DKO embryos. Cardiomyocytes are co-labeled by immunostaining of cTnT antibodies. *p < 0.05, **p < 0.01, ***p < 0.001 by unpaired Student’s *t*-test.

Next, EdU labeling revealed that loss of *Yap*/*Taz* markedly inhibited endothelial cell proliferation (Fig. 4A,B). In addition, the DKO embryos exhibited decreased and increased cardiomyocyte proliferation in compact and trabecular myocardium respectively in both left and right ventricles (Fig. 4C–E and Suppl. Fig. 5), which further substantiated the ventricular noncompaction phenotype in DKO embryos. It was worth to note that there was no TUNEL positive cells in ventricular free walls in both control and DKO hearts while positive signals were equally present in atrioventricular canal cushions (Fig. 4F), suggesting that deletion of *Yap/Taz* in endocardial lineage does not affect cell apoptosis. Collectively, these results demonstrate that *Yap/Taz* is required for endothelial cell proliferation during coronary artery development, and suggest an imbalanced cardiomyocyte proliferation between trabecular and compact myocardium leading to ventricular noncompaction.

### Deletion of *Yap/Taz* downregulates the expression of *Tm4sf1*

**Fig. 5:**
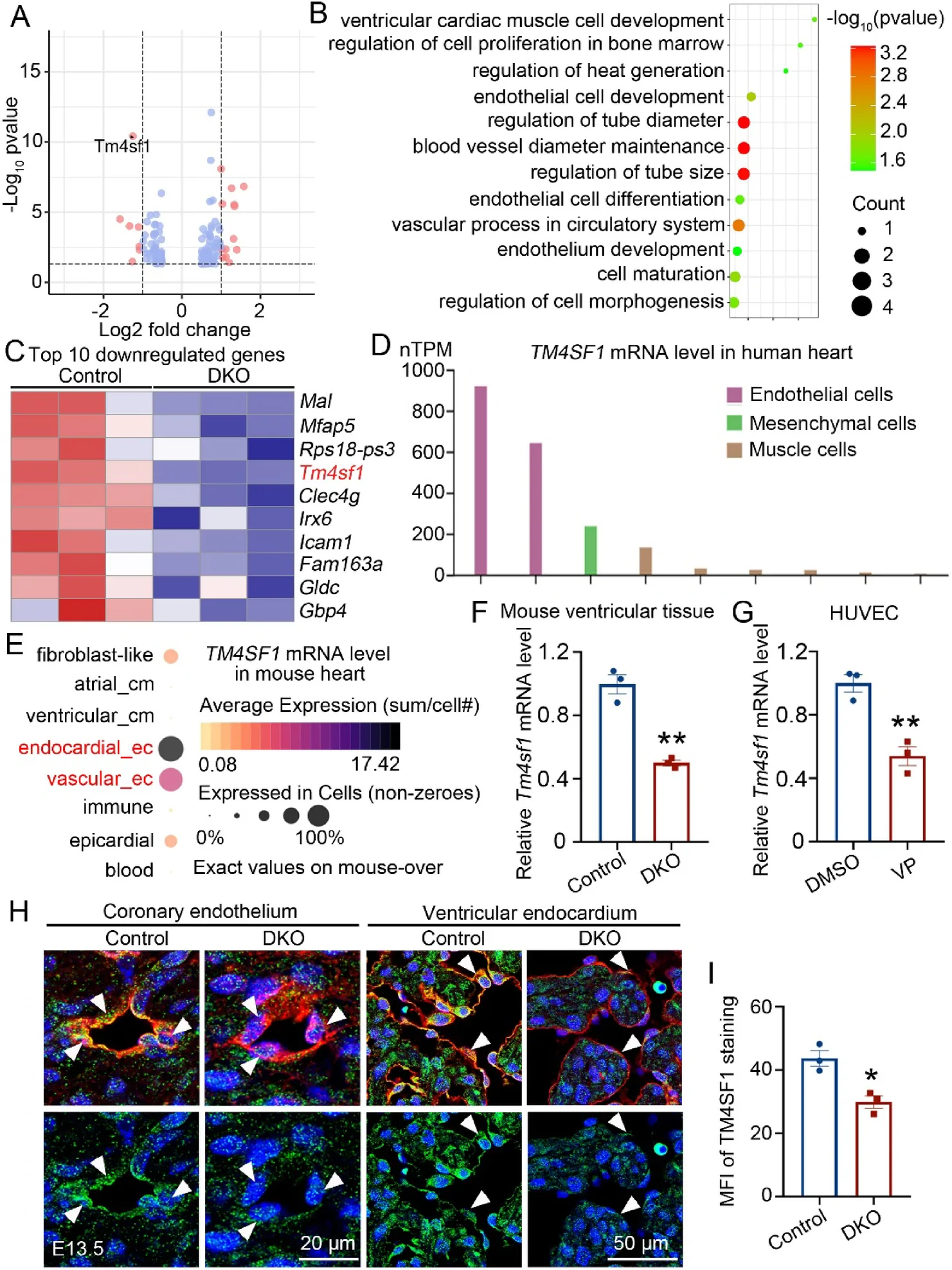
Loss of YAP/TAZ inhibits TM4SF1 expression in endothelial cells. (**A**–**C**) Ventricular heart tissues were isolated from control and DKO embryos at E12.5 and subjected to RNA‑seq analysis. (**A**) Volcano plot showing differentially expressed genes between the two groups. (**B**) Bubble plot of GO enrichment analysis for biological processes associated with downregulated genes. (**C**) Heatmap displaying the top 10 downregulated genes. (**D**, **E**) Public single cell RNA-seq datasets (GSE109816 and GSE193346) illustrate the expression profile of *TM4SF1* across cardiac cell types in human (**D**) and mouse (**E**) hearts. (**F**, **G**) qPCR analysis of *Tm4sf1* expression in ventricular tissues from E12.5 control and DKO embryos (**F**) and in HUVECs treated with verteporfin (VP, 1 µM) or DMSO for 12 h (**G**). *n* = 3/group. (**H**, **I**) Representative immunofluorescence images (**H**) and quantitative analysis of TM4SF1 mean fluorescence intensity (MFI) (**I**) in endothelial cells marked by VE‑cadherin (VE-Cad). *n* = 3/group. *p < 0.05, **p < 0.01 by unpaired Student’s *t*-test.

To identify downstream effectors of *Yap/Taz* in regulation of coronary artery development, we then harvested heart ventricular tissues from E12.5 control and DKO embryos for transcriptomic profiling. Loss of *Yap/Taz* resulted in 137 differentially expressed genes (Fig. 5A). Among them, 73 and 64 genes were upregulated and downregulated (Fig. 5A), respectively. Gene Ontology enrichment analysis revealed that the downregulated transcripts were involved in regulation of tube diameter, endothelium development, and regulation of heart generation (Fig. 5B). The top 10 downregulated genes were involved in endothelial cell differentiation and regulation of tube diameter, while the top 10 upregulated genes were associated with tissue homeostasis and phagocytic vesicle (Suppl. Fig. 6). Among the top 10 downregulated genes, *Tm4sf1* was found to be expressed exclusively in endothelial cells in both human and mouse hearts (Fig. 5C–E), according to published single-cell RNA-seq datasets. Knockout of *Tm4sf1* in mice is known to result in embryonic lethality at mid-gestation due to impaired vasculature development^32^. In addition, *Tm4sf1* has been shown to critically regulate endothelial migration and tumor angiogenesis^33–36^. These observations led us to hypothesize that TM4SF1 may function as a downstream target of *Yap/Taz* in coordination of coronary artery formation. The reduction of *Tm4sf1* transcripts in DKO hearts was then validated by qPCR (Fig. 5F). Verteporfin is a well-established YAP inhibitor that binds YAP, promotes its cytoplasmic retention, and facilitates its proteasomal degradation, thereby preventing YAP nuclear translocation and TEAD-mediated transcription^37^. Consistent with the in vivo data, treatment of HUVECs with verteporfin similarly attenuated *TM4SF1* mRNA abundance (Fig. 5G). Moreover, immunofluorescence revealed markedly diminished TM4SF1 protein expression in endocardial and coronary endothelial cells of DKO embryos (Fig. 5H,I and Suppl. Fig. 7). Together, the results of gene expression analysis indicate that *Tm4sf1* may function downstream of YAP/TAZ to regulate coronary artery development.

### YAP mediates VEGF-induced upregulation of TM4SF1 in endothelial cells

**Fig. 6:**
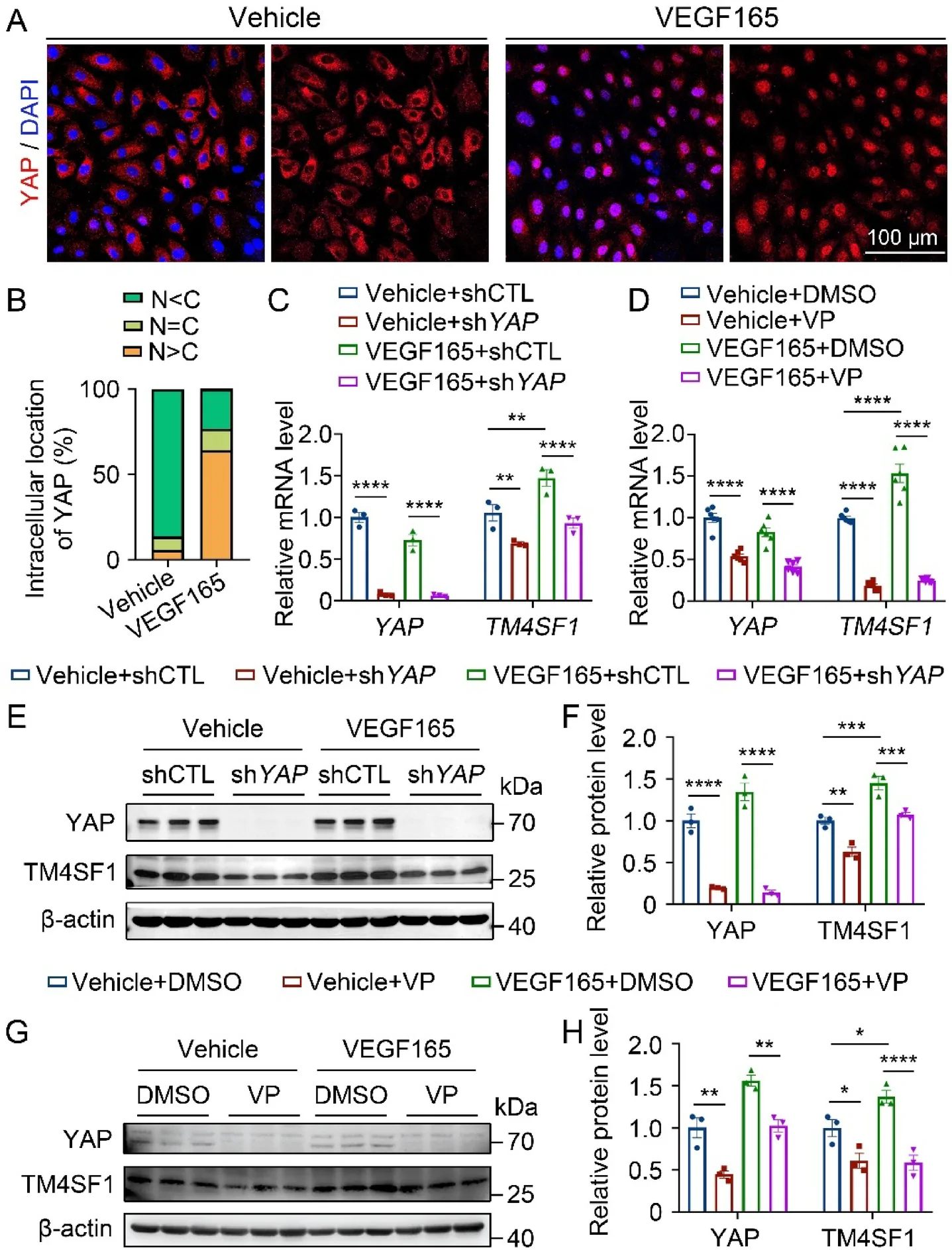
YAP mediates VEGF-induced upregulation of TM4SF1 in endothelial cells. (**A**, **B**) HUVECs were stimulated with VEGF165 (50 ng/ml) for 2 h. Immunofluorescence images (**A**) and quantitative analysis (**B**) show the subcellular localization of YAP. *n* = 4/group. N, nucleus; C, cytoplasm. (**C**, **E**, **F**) HUVECs were transduced with control or sh*YAP1* lentivirus, followed by stimulation with or without VEGF165. Expression of *YAP1* and *TM4SF1* was analyzed by qPCR (**C**) and Western blot (**E**, **F**). *n* = 3/group. (**D**, **G**, **H**) HUVECs were treated with verteporfin (VP, 1 µM) or DMSO, and followed by stimulation with or without VEGF165. Expression of *YAP1* and *TM4SF1* was measured by qPCR (**D**, *n* = 6/group) and Western blot (**G**, **H**, *n* = 3/group). *p < 0.05, **p < 0.01, ***p < 0.001, ****p < 0.0001 by two-way ANOVA.

Given that YAP/TAZ are major mediators of VEGF-induced angiogenetic gene programs in endothelial cells, we next investigated whether TM4SF1 is a potential target in this pathway. In line with the literatures^31^, we firstly confirmed that VEGF treatment induced significant nuclear accumulation of YAP in HUVECs (Fig. 6A,B). VEGF induced significant elevation of *TM4SF1* transcripts, and this action was attenuated by siRNA-mediated silencing of *YAP* or pharmacological inhibition of YAP signaling (Fig. 6C,D). This finding was validated at the protein level (Fig. 6E–H). However, overexpression of YAP failed to upregulate *TM4SF1* transcripts under basal conditions or upon VEGF stimulation (Suppl. Fig. 8). These findings indicate that YAP is required, but not sufficient, for VEGF-induced upregulation of TM4SF1 in endothelial cells.

### TM4SF1 mediates VEGF-driven angiogenesis downstream of YAP

**Fig. 7:**
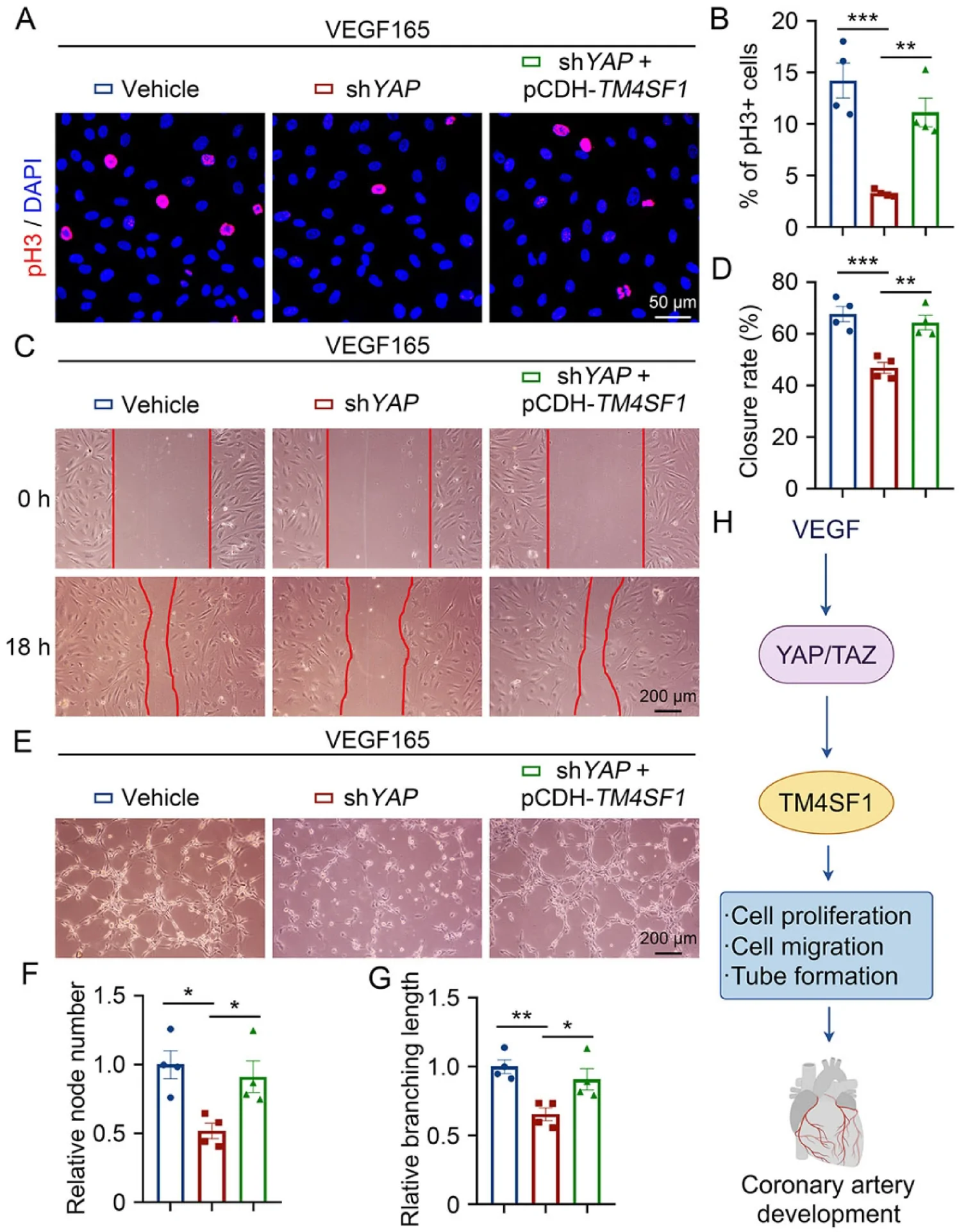
TM4SF1 acts downstream of YAP to mediate VEGF-driven angiogenesis. (**A**, **B**) HUVECs were stimulated with VEGF165 (50 ng/ml) and transduced with control lentivirus, shYAP1 lentivirus, or sh*YAP1* plus pCDH-*TM4SF1* rescue lentivirus. Cell proliferation was assessed by immunofluorescence staining for phospho-histone H3 (pH3). Representative images (**A**) and quantification of the percentage of pH3‑positive cells (**B**) are shown. *n* = 4/group. (**C**, **D**) Representative images from a scratch wound‑healing assay performed under the indicated conditions (**C**) and the corresponding quantification of wound closure rate (**D**) *n* = 4/group. (**E**–**G**) Representative images of in vitro tube formation assays under the indicated treatments (**E**). Quantification of the number of branching points (**F**) and total tube length (**G**) is shown. *n* = 4/group. (**H**) Schematic model illustrating that TM4SF1 functions downstream of YAP/TAZ to mediate VEGF‑driven coronary artery development. This figure was drawn with Figdraw (https://www.figdraw.com). *p < 0.05, **p < 0.01, ***p < 0.001 by one-way ANOVA.

We next employed angiogenesis assays in HUVECs to investigate whether TM4SF1 acts downstream of YAP to mediate VEGF-driven angiogenesis. pH3 immunostaining revealed that *YAP* knockdown with lentivirus significantly inhibited cell proliferation, while this inhibition was reversed by *TM4SF1* overexpression (Fig. 7A,B). Similarly, YAP‑deficient cells exhibited markedly delayed wound closure in a scratch‑migration assay, and *TM4SF1* overexpression effectively rescued the migratory capacity (Fig. 7C,D). In an in vitro tube‑formation assay, *YAP* knockdown severely compromised the angiogenic potential of HUVECs, as reflected by reduced branching points and total tube length; both parameters were significantly recovered upon *TM4SF1* overexpression (Fig. 7E–G). Collectively, these results demonstrate that TM4SF1 is a crucial downstream target of YAP/TAZ in mediating VEGF-driven angiogenesis.

## Discussion

Despite YAP/TAZ are well established mediators of VEGF-driven angiogenesis, their downstream targets remain incompletely defined. In this study, by using genetic mice models, transcriptomic profiling and in vitro angiogenesis assays, we demonstrate that TM4SF1 as a novel downstream mediator of YAP/TAZ involved in coronary artery development (Fig. 7H).

The endocardium is a major source of coronary arterial endothelial cells but contributes minimally to coronary venous endothelium^6,12,38^. To determine the cell-autonomous role of endothelial YAP/TAZ in coronary artery development, we employed *Nfatc1*^Cre^ to selectively delete *Yap/Taz* in endocardial lineage. Consistent with previous reports by *Artap et al.*^29^, loss of YAP/TAZ in this lineage markedly impaired myocardial growth and led to early postnatal lethality. Moreover, previous studies have demonstrated that endocardial YAP/TAZ promotes cardiomyocyte proliferation via NRG1-mediated paracrine signaling^29^. Although earlier studies did not report obvious coronary vascular defects in these mutants^29^, the ventricular noncompaction phenotype and necrotic lesions observed in the ventricular septum of DKO embryos prompted us to re-examine coronary development. First, hypoxia probe labeling revealed increased hypoxia in DKO embryonic hearts compared to controls, suggesting impaired coronary circulation. This observation was confirmed by whole-mount staining, which showed severe coronary artery defects in DKO embryos, including the absence of major coronary arteries. Notably, coronary vein development was comparable between control and DKO embryos. This artery-specific phenotype aligns with the known predominant contribution of the endocardium to arterial rather than venous endothelial cells^5,6^. Beyond the established roles of YAP/TAZ in cardiomyocyte proliferation^25^, atrioventricular cushion formation^27^, and epicardial development^28^, our study reveals a new cell-autonomous function of endothelial YAP/TAZ in coronary artery development. Future studies using inducible, endothelial-specific deletion models (e.g., Cdh5-CreERT2) will be required to dissect the direct versus indirect contributions of YAP/TAZ to early coronary angiogenesis and later artery formation.

Although YAP/TAZ are known to initiate angiogenic gene programs in response to VEGF^31^, their downstream targets in developing coronary endothelial cells remain unclear. By using transcriptomic profiling, we found that TM4SF1 expression in coronary endothelial cells is highly dependent on YAP/TAZ. This in vivo observation was confirmed by the findings that YAP/TAZ is dispensable for VEGF-induced upregulation of TM4SF1 in HUVECs. However, YAP overexpression alone failed to elevate TM4SF1 expression under basal conditions or upon VEGF stimulation, suggesting that additional co-factors are required for YAP-dependent TM4SF1 transcription, a topic warranting further investigation. Future studies are also needed to determine whether *TM4SF1* is a direct transcriptional target of YAP/TAZ.

TM4SF1 is a well-established regulator of both developmental and pathological angiogenesis^33–36^. Global knockout of *Tm4sf1* has been reported to cause embryonic lethality by E9.5 due to impaired vascular development^32^. TM4SF1 deficiency has been shown to inhibit endothelial cell proliferation, migration, and tube formation in vitro and to impair pathological angiogenesis in vivo^35^^36^. Consistent with these documented roles of TM4SF1 in regulating the angiogenic capacity of endothelial cells, our in vitro tube formation assays demonstrated that TM4SF1 overexpression rescued the angiogenic defects induced by YAP inhibition. Although these findings collectively suggest a potential role for TM4SF1 in coronary artery development, functional validation through genetic knockout studies in animal models remains essential.

In summary, we identify a novel VEGF/YAP/TM4SF1 regulatory axis that is crucial for coronary artery formation. This finding advances our fundamental understanding of cardiac development and refines the model of coronary vessel specification. Future studies should investigate whether TM4SF1 expression or activity is altered in congenital heart defects associated with coronary malformations and evaluate its potential as a therapeutic target for promoting coronary angiogenesis in ischemic heart disease.

## Materials and methods

### Animals

*Yap*^f/f^*/Taz*^f/f^ mouse line was kindly provided by professor Shengpeng Wang at Xi’an Jiaotong University. All animals were housed under specific pathogen-free (SPF) conditions in a temperature- and humidity-controlled Animal Research Center of Xi’an Jiaotong University. *Yap*^f/f^*/Taz*^f/f^ mice were crossed with *Nfatc1*^cre^ mice to obtain endocardial lineage-specific *Yap/Taz* knockout mice. Noontime on the day of detecting vaginal plugs was designated as embryonic day (E) 0.5. Mice were euthanized in a gradual-fill carbon dioxide chamber (fill rate of approximately 30% chamber volume per minute). Following respiratory arrest, death was confirmed by cervical dislocation performed by trained personnel before tissue collection. All experimental protocols were approved by the Institutional Animal Care and Use Committee (IACUC) of Xi’an Jiaotong University and adhered to the Guidelines for the Care and Use of Laboratory Animals (National Institutes of Health).

### Histology and immunostaining

Heart tissues were fixed in 4% PFA after freshly harvested. After three washes with PBS and dehydrated through a graded ethanol series, cleared in xylene, the tissues were embedded in paraffin. Sections were cut at a thickness of 5 μm. Hematoxylin and eosin (H&E) staining was performed according to standard protocols. For immunofluorescence staining, paraffin sections were deparaffinized in xylene and rehydrated through a descending ethanol series. After antigen retrieval, sections were blocked for 1 h in 5% horse serum (Solarbio, SL042) at room temperature. Primary antibodies (Supplementary Table S1) were incubated at 4 °C overnight, followed by incubation with corresponding secondary antibodies (Supplementary Table S1) at room temperature for 1 h. Nuclei were counterstained with DAPI (Beyotime, C1006) for 5 min. All images were acquired using a confocal microscope (Olympus FV3000 or Leica STELLARIS 5). Quantitative image analysis was conducted using ImageJ software.

### Detection of myocardial hypoxia

Hypoxia was assessed in embryonic heart tissues using the Hypoxyprobe™ kit (Hypoxyprobe Inc.). Pregnant mice were intraperitoneally (ip) injected with Hypoxyprobe™-1 at a dose of 60 mg/kg. After 90 min, embryonic hearts were dissected and fixed in 4% PFA for 2 h at 4 °C. Following fixation, tissues were washed three times with PBS, sequentially cryoprotected in 15% and 30% sucrose solutions, and embedded in OCT compound. Hypoxic regions were subsequently detected via immunofluorescence staining using the Hypoxyprobe monoclonal antibody followed by an Alexa Fluor 488-conjugated goat anti-mouse IgG secondary antibody. DAPI (Beyotime, C1006) was applied to counterstain nuclei for 5 min.

The HP1 + area was quantified using ImageJ software. Briefly, images of HP1‑immunostained sections were captured under identical exposure settings across all groups. The HP1 + signal was thresholded to remove background noise, and the positive area was measured within the entire myocardial region.

### Whole-mount staining

Embryonic heart tissues were isolated and fixed in 4% paraformaldehyde (PFA) for 6 h at 4 °C. After fixation, tissues were washed three times with PBS for 15 min each under gentle shake. Under a stereomicroscope, each heart was bisected sagittally to separate the venous region (dorsal region) from the arterial region (ventral region). To obtain a flat internal surface, portions of the interventricular septum and trabeculae were carefully removed. Additional trimming included excision of part of the right ventricle and endocardial cushion tissue within the arterial region. For immunofluorescence staining, the prepared tissues were blocked with 10% donkey serum (Solarbio, SL042) at room temperature for 2 h. Primary antibody incubation was performed overnight at 4 °C with continuous gentle shaking (refer to Supplementary Table S1 for antibody details). Following primary incubation, samples were washed six times with 0.3% PBST (PBS containing 0.3% Triton X-100). Subsequently, tissues were incubated overnight at 4 °C with appropriate fluorescent secondary antibodies (Suppl. Table S1) under shaking. After six additional washes with 0.3% PBST (PBS containing 0.3% Triton X-100), tissues were mounted using an antifade mounting medium for fluorescence preservation and imaging.

Vessel density was quantified using ImageJ software. Coronary artery density was determined as the percentage of PECAM1 + ICAM2+ area normalized to the left ventricular area. Coronary vein density was determined as the percentage of EMCN+ area normalized to the total heart area.

### Cell proliferation and apoptosis assays

For cell proliferation analysis, pregnant mice were intraperitoneally injected with 5-ethynyl-2′-deoxyuridine (EdU; Life Technologies) at a dosage of 100 mg/kg. Embryonic hearts were harvested 2 h post-injection and processed into paraffin sections as described in the Immunostaining section above. EdU staining was subsequently performed following the manufacturer’s protocol, and Nuclei were labeled with DAPI for 5 min as a counterstain. Apoptotic cells were visualized in developing embryonic hearts using the DeadEnd™ Fluorometric TUNEL kit (Promega, G3250) according to the manufacturer’s instructions. Following the TUNEL reaction, sections were counterstained with DAPI to label all nuclei.

### Cell culture

Human umbilical vein endothelial cells (HUVECs) were maintained in 0.1% gelatin coated plates and cultured in endothelial cell-specific growth medium (Sciencell, 1001) enriched with 5% fetal bovine serum, along with 100 U/ml of penicillin and streptomycin. All cells were cultured at 37 °C in a humidified cell incubator supplied with 5% CO₂ and normoxic (21% O₂) atmosphere.

### Immunofluorescence on HUVECs

Cover slips were pre-placed in a 24-well plate and coated with 0.1% gelatin. Cells were then seeded into the 24-well plate and subjected to the corresponding treatments. After treatment, cells were fixed with 4% PFA for 15 min, washed three times with PBS, and permeabilized with 0.5% PBS containing Triton X-100 (PBST) for 10 min. After three additional PBS washes, cells were blocked with 3% BSA at room temperature for 1 h. Primary antibody incubation was performed overnight at 4 °C. Following three PBS washes, cells were incubated with a secondary antibody at room temperature for 1 h. Finally, nuclei were counterstained by incubation with DAPI.

### Cell migration assays

HUVECs transduced with lentivirus were seeded into 12-well plates and cultured until reaching 80–90% confluence the following day. The cells were then maintained in medium containing 50 ng/mL VEGF165 (R&D, 293-VE-050/CF) and 2% serum. A scratch wound was introduced across the monolayer using a 200ul sterile pipette tip. The wounded areas were photographed immediately after scratching (0 h) and again after 18 h of incubation. The wound healing rate was quantified by measuring the change in the scratch area using ImageJ software.

### Tube formation assays

For the tube formation assay, each well of a 96-well plate was pre-coated with 50 µL of Matrigel (CORNING, 354236). Subsequently, 1 × 10⁴ lentivirus-transduced HUVECs were seeded per well. The cells were cultured in medium containing 2% serum and 50 ng/mL VEGF165. Tube formation was allowed to proceed for 4 h, after which representative images were captured for analysis.

### Western blot

HUVECs were lysed using RIPA buffer supplemented with protease inhibitors. The lysates were centrifuged at 12,000 rpm at 4 °C for 15 min, and the supernatant was collected. Protein concentration was quantified using a BCA assay kit, followed by denaturation with SDS loading buffer. Proteins were separated by SDS-PAGE (sodium dodecyl sulfate–polyacrylamide gel electrophoresis) and subsequently transferred onto a PVDF membrane. The membrane was blocked at room temperature for 1 h with 5% BSA prepared in TBST. Primary antibody incubation was performed overnight at 4 °C on a shaking platform using antibodies diluted in 5% BSA/TBST. After washing three times with TBST, the membrane was incubated with secondary antibody (diluted in 5% BSA/TBST) for 1 h at room temperature. Following three additional TBST washes, protein signals were detected using an ECL reagent kit.

### RNA extraction and quantitative real-time PCR (qPCR)

Total RNA was extracted from embryonic ventricular tissues or HUVECs using Trizol reagent. RNA samples were reverse transcribed into cDNA using a HiScript II reverse transcriptase Kit (Vazyme R222). Quantitative real-time PCR (qPCR) was then performed using SYBR Green PCR Master Mix (Genstar A304-05) and gene-specific primers (Suppl. Table S2). Results were standardized by GAPDH.

### RNA-seq analysis

Heart ventricular tissues were harvested from control and DKO embryos at E12.5 for RNA sequencing. Sequencing reads generated on the Illumina platform were quality‑assessed and filtered using Fastp (v0.23.1)^39^ with the following exclusion criteria: (1) either read in a pair contained adapter contamination; (2) more than 10% of bases were undetermined in either read; (3) more than 50% of bases in either read were of low quality (Phred score < 5). The resulting clean reads were aligned to the mouse reference genome (GRCm39) using Hisat2 (v2.0.5)^40^. Differential expression analysis was performed with the DESeq2 package (R 4.2.1)^41^. Genes with fewer than 50 counts across all samples were filtered out. Significantly differentially expressed genes (DEGs) were defined as those with an adjusted p‑value (padj) ≤ 0.05 and |log2(fold change)| ≥0.5. Gene Ontology (GO) were conducted using the clusterProfiler package (R 4.5.2). GO terms with an adjusted *p*‑value < 0.05 were considered significantly enriched.

### scRNA-seq data analysis

Human single-cell RNA sequencing (scRNA-seq) data were retrieved from the Protein Atlas portal (version 22, v22.proteinatlas.org), which includes the dataset (GSE109816) published by *Wang et al.*^42^. Mouse single-cell data (GSE193346) were obtained from the study by *Feng et al.*^43^. Processed data from the latter publication are publicly available through the UCSC Cell Browser at: https://cells-test.gi.ucsc.edu/?ds=mouse-dev-heart.

### Statistics

All data are presented as the mean ± SEM. Statistical analyses were conducted using GraphPad Prism software. Differences between two groups were assessed using the Student’s *t*-test, while comparisons among multiple groups were performed by one-way ANOVA followed by Tukey’s post‑hoc test. A *p*‑value < 0.05 was considered statistically significant.

## Supplementary Information

Below is the link to the electronic supplementary material.

Supplementary Material 1

Supplementary Material 2

Supplementary Material 3

## Data Availability

The data associated with this paper are available upon reasonable request to the corresponding authors. The RNA-sequencing data has been deposited in the National Genomics Data Center, China National Center for Bioin-formation under the accession code CRA039344.
