# Supplementary material for: TM4SF1 mediates YAP/TAZ regulation of coronary artery formation
Source: Sci Rep. 2026 Jun 11;16:26625. doi: 10.1038/s41598-026-57461-x (PMC13503703; doi:10.1038/s41598-026-57461-x)
Supplement: Supplementary file 1 [file 41598_2026_57461_MOESM1_ESM.pdf]

Figure 6E

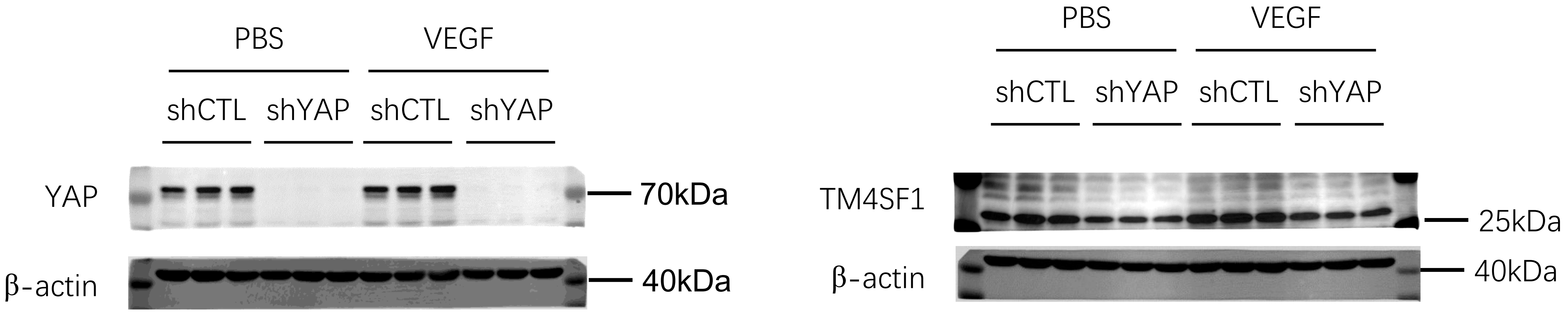
PBS
VEGF
shCTL
shYAP
shCTL
shYAP
YAP
70kDa
β-actin
40kDa
PBS
VEGF
shCTL
shYAP
shCTL
shYAP
TM4SF1
25kDa
β-actin
40kDa

Figure 6G

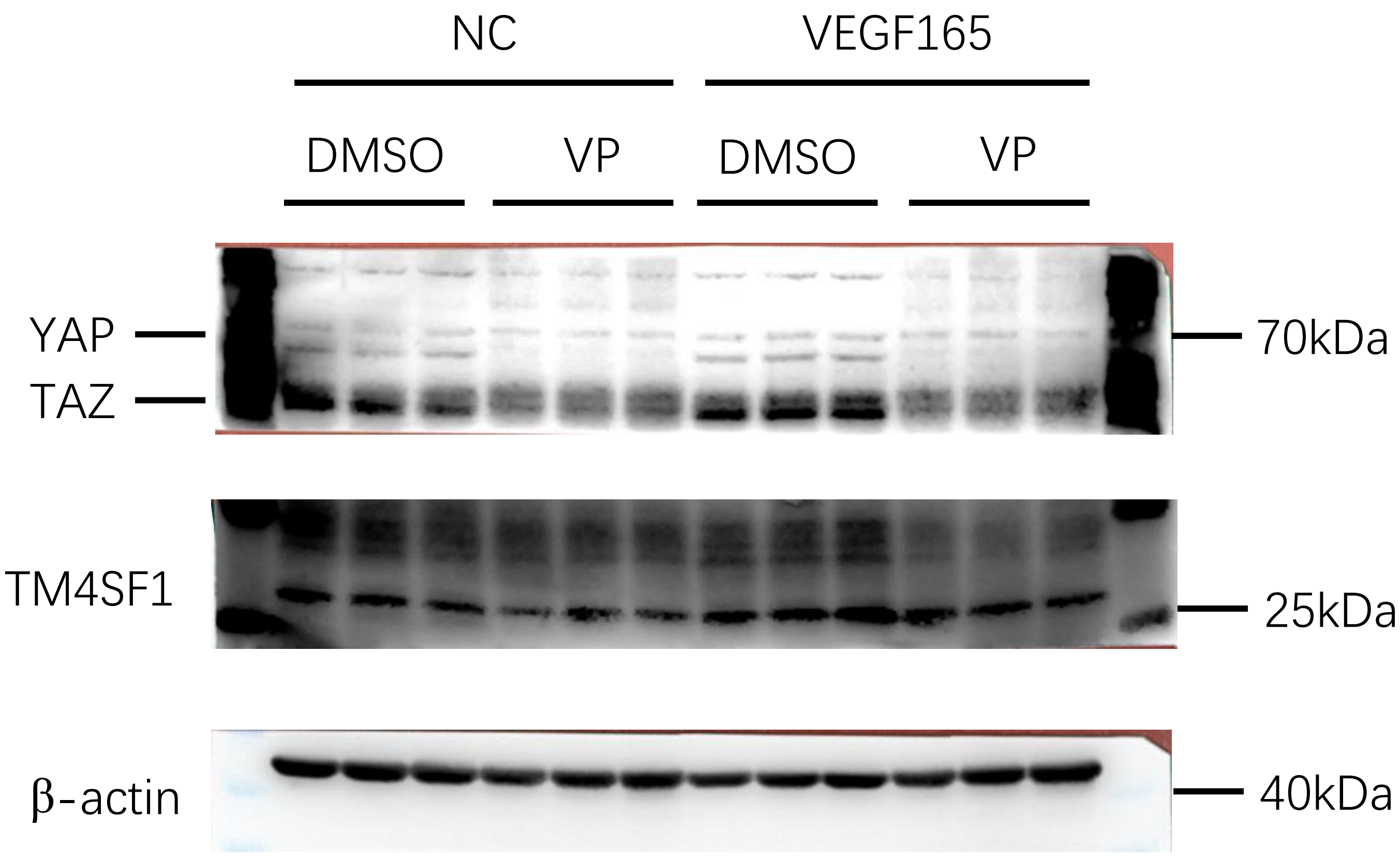
NC
VEGF165
DMSO
VP
DMSO
VP
YAP
TAZ
70kDa
TM4SF1
25kDa
β-actin
40kDa
