# Supplementary material for: TM4SF1 mediates YAP/TAZ regulation of coronary artery formation
Source: Sci Rep. 2026 Jun 11;16:26625. doi: 10.1038/s41598-026-57461-x (PMC13503703; doi:10.1038/s41598-026-57461-x)
Supplement: Supplementary file 2 [file 41598_2026_57461_MOESM3_ESM.docx]

**Supplementary Figure 1. Knockout efficiency of YAP and TAZ in the right ventricle.**

Immunofluorescence staining showing deletion efficiency of YAP (green, **A**) and TAZ (green, **B**) in the right ventricular region of hearts from DKO embryos (*Yap*^f/f^:*Taz*^f/f^: *Nfatc1*^Cre^) and controls (*Yap*^f/f^:*Taz*^f/f^ or *Yap*^f/+^:*Taz*^f/f^) at embryonic day 13.5 (E13.5). Endocardial cells (arrowhead) and coronary arterial endothelial cells (arrow) are identified by VE-Cadherin co-staining (red).

**Supplementary Figure 2. All replicates of H&E staining related to Figure 2A.**

Hematoxylin and eosin (H&E) staining of heart sections from control and DKO embryos at E13.5.

**Supplementary Figure 3. YAP/TAZ deficiency causes right ventricular noncompaction.**

(**A**) Immunostaining of EMCN was performed to label the right ventricular trabecular myocardium (TM) and compact myocardium (CM) in control and DKO embryos at the indicated developmental stages. The widths of the TM (indicated by white double‑headed arrows) and the CM (indicated by yellow lines) were quantified and are presented as the CM/TM ratio (**B**). n = 4-5/group. **p* < 0.05, ***p* < 0.01 by unpaired Student’s *t*-test.

**Supplementary Figure 4.** **All replicates of whole-mount staining related to Figure 3C and 3F.**

(**A**, **B**) Whole-mount staining of E15.5 hearts with antibodies against DLL4 (arterial endothelial marker), PECAM1 and ICAM2 (pan-endothelial markers), EMCN (venous endothelial marker) and ERG (endothelial nuclei marker).

**Supplementary Figure 5. Loss of YAP/TAZ impairs cell proliferation.**

(**A**-**C**) Representative immunofluorescence images (**A**) and quantitative analysis of proliferating cardiomyocytes positive for EdU (**B**) within right ventricular region of the heart sections from E13.5 control and DKO embryos. Endocardial cells were marked by VE-cadherin staining. Cardiomyocyte proliferation was quantified separately in the compact myocardium (CM) and trabecular myocardium (TM) (**B**). The ratio of cell proliferation in CM to that in TM is shown in (**C**). RV, right ventricle. (**D**) Comparison of cardiomyocyte proliferation between left and right ventricles. n = 4/group. ***p* < 0.01; *****p* < 0.0001 by unpaired Student’s *t*-test (**B** and **C**) and by two-way ANOVA (**D**).

**Supplementary Figure 6. GO enrichment analysis of differentially expressed genes caused by loss of YAP/TAZ.**

(**A**) Bubble plot of GO enrichment analysis for biological processes associated with upregulated genes. (**B**) Heatmap displaying the top 10 upregulated genes. (**C** and **D**) tables showing the functional categories of top 10 upregulated and top 10 downregulated genes belong to.

**Supplementary Figure 7. Low magnification images of TM4SF1 staining related to Figure 5H.**

Representative images of immunofluorescence staining showing TM4SF1 expression in coronary endothelial cells (arrows) and ventricular endocardial cells (arrowheads), as identified by co-staining with VE-cadherin (VE-Cad) antibodies.

**Supplementary Figure 8. YAP overexpression alone does not elevate *TM4SF1* transcripts.**

HUVECs were treated with Ad-YAP or Vehicle under stimulation with or without VEGF165. Expression of *YAP1* and *TM4SF1* was measured by qPCR. n = 5/group. **p* < 0.05, *****p* < 0.0001 by two-way ANOVA.

**Supplementary Table S1. A list of antibodies used in this study**

| Antibody | Company | Cat# | Source | WB dilution | IF dilution |
| --- | --- | --- | --- | --- | --- |
| YAP | Abcam | ab205270 | Rabbit | 1:2000 | 1:300 |
| TAZ | Proteintech | 23306-1-AP | Rabbit | 1:1000 | 1:300 |
| VE-cadherin | R&D | AF1002 | Goat | N/A | 1:400 |
| EMCN | Santa | sc-53941 | Rat | N/A | 1:200 |
| PECAM1 | BD Biosciences | 553370 | Rat | N/A | 1:200 |
| ICAM2 | BD Biosciences | 553325 | Rat | N/A | 1:200 |
| DLL4 | R&D | AF1389 | Rabbit | N/A | 1:200 |
| ERG | Proteintech | 14356-1-AP | Rabbit | N/A | 1:400 |
| VEGFR3 | R&D | AF743 | Rabbit | N/A | 1:400 |
| TM4SF1 | NOVUS | NBP1-76549 | Rabbit | 1:1000 | 1:400 |
| ꞵ-actin | Proteintech | 205536-AP | Rabbit | 1:1000 | N/A |
| pH3 | CST | 9701S | Rabbit | N/A | 1:400 |
| cTnT | Proteintech | 15513-1-AP | Rabbit | N/A | 1:200 |

**Supplementary Table S2. A list of primers used in this study**

| **Human gene** | **Forward sequence** | **Reverse sequence** |
| --- | --- | --- |
| *YAP1* | TAGCCCTGCGTAGCCAGTTA | TCATGCTTAGTCCACTGTCTGT |
| *TM4SF1* | GGCTACTGTGTCATTGTGGCAG | ACTCGGACCATGTGGAGGTATC |
| *GAPDH* | CTCCTCACAGTTGCCATGTA | GTTGAGCACAGGGTACTTTATTG |
| **Mouse gene** | **Forward sequence** | **Reverse sequence** |
| *Tm4sf1* | GGATGAAGAGGACTGCTGTGGT | CCACGATTCCAATCAGAGCAGC |
| *Gapdh* | TGACCTCAACTACATGGTCTACA | CTTCCCATTCTCGGCCTTG |
